# Neither low social support nor low decision latitude at work is associated with disease remission among patients with rheumatoid arthritis: results from the Swedish EIRA study

**DOI:** 10.1186/s13075-022-02892-w

**Published:** 2022-08-23

**Authors:** Louise Hedenstierna, Anna Karin Hedström, Lars Klareskog, Daniela Di Giuseppe, Lars Alfredsson, Johan Askling, Sofia Ernestam, Saedis Saevarsdottir, Lotta Ljung

**Affiliations:** 1 Department of Medicine, Solna, Karolinska Institutet, Stockholm, Sweden; 2 Academic Specialist Center, Stockholm Health Services, Stockholm, Sweden; 3 Institute of Environmental Medicine, Karolinska Institutet, Stockholm, Sweden; 4 Center for Rheumatology, Karolinska Institutet, Solnavägen 1E, 102 35 Stockholm, Sweden; 5 Department of Clinical Neuroscience, Karolinska Institutet, Stockholm, Sweden; 6 Center for Occupational and Environmental Medicine, Region Stockholm, Stockholm, Sweden; 7 Rheumatology, Theme Inflammation & Ageing, Karolinska University Hospital, Stockholm, Sweden; 8 Faculty of Medicine, School of Health Sciences, University of Iceland, Reykjavik, Iceland; 9 Department for Public Health and Clinical Medicine/Rheumatology, Umeå University, Umeå, Sweden

## Abstract

**Objectives:**

To investigate the association between psychosocial vulnerability, defined as either low social support or low decision latitude at work, and disease remission at 3, 12, and 60 months in patients with rheumatoid arthritis (RA).

**Methods:**

This cohort study included all patients enrolled in both the Swedish Epidemiological Investigation of Rheumatoid Arthritis (EIRA) 1996–2015 and the Swedish Rheumatology Quality Register (SRQ, *n* = 2820). Information on social support and decision latitude at work at RA diagnosis were identified from the EIRA questionnaire. Indexes for levels of social support and decision latitude at work, respectively, were calculated based on the questionnaire. Low social support and low decision latitude at work, respectively, were identified by a score in the lowest quartile and compared with the three other quartiles (not low). Disease-activity parameters were retrieved from SRQ at 3, 12, and 60 months. The associations between social support or decision latitude at work, respectively, and Disease Activity Score 28 joint count with C-reactive protein (DAS28-CRP) remission were analysed using logistic regression models adjusted for age, sex, smoking habits, alcohol habits, symptom duration, and educational level.

**Results:**

Having low social support (*n* = 591) was not associated with DAS28-CRP remission at 3 (OR 0.93, 95% CI 0.74–1.16), 12 (OR 0.96, 95%CI 0.75–1.23), or 60 (OR 0.89, 95%CI 0.72–1.10) months compared to not low social support (*n* = 2209). No association was observed for low (*n* = 212) versus not low (*n* = 635) decision latitude at work and DAS28-CRP remission at 3 (OR 0.84, 95%CI 0.54–1.31), 12 (OR 0.81, 95%CI 0.56–1.16), or 60 (OR 1.37, 95%CI 0.94–2.01) months.

**Conclusion:**

In a country with general access to healthcare, psychosocial vulnerability does not influence the likelihood of achieving remission in early RA.

**Supplementary Information:**

The online version contains supplementary material available at 10.1186/s13075-022-02892-w.

## Introduction

Rheumatoid arthritis (RA) is a chronic systemic inflammatory disease characterized by synovial inflammation, pain, and fatigue [1, 2]. The disease increases the risk of structural joint destruction and subsequently impaired joint function, leading to decreased working ability among affected individuals, with impaired life income for patients and costs for society [3, 4]. To minimize the consequences of inflammation in RA, the current clinical strategy is a targeted approach including early diagnosis and treatment aiming for remission or, when remission is not achievable, low disease activity [5]. However, some patients with RA do not reach the treatment goals; thus, there is a need for further insights into how contextual factors, including environmental and personal factors, influence patient prognosis.

Previous studies on psychosocial factors in RA have mainly focused on the association with the risk of RA development and found that contextual factors including low educational level and low decision latitude at work were associated with increased risk of the disease [6–8]. The concept of decision latitude at work was introduced by Karasek in 1990 [9]. The theory behind the concept suggests that a low level of influence on the daily work situation (“low decision latitude”) would be a marker of long-term stress [10]. Questions on decision latitude at work were introduced in the EIRA questionnaire in 1996.

While some studies have investigated the influence of psychosocial factors such as social deprivation and educational level on disease outcome [11, 12], one of which from the Epidemiological Investigation of Rheumatoid Arthritis (EIRA) study [13], to our knowledge there is limited evidence on the impact of psychosocial factors, such as the level of social support and decision latitude at work on outcome in RA.

Improved knowledge of the baseline predictors of response would facilitate rational use of the therapeutic arsenal, not only drugs but also interventions such as psychological support, thereby improving the chance for good outcomes for individual patients.

This study therefore aimed to increase our understanding of the impact of psychosocial vulnerability on RA disease outcome by analysing the association between low social support outside work, low decision latitude at work, and disease remission in the Swedish setting, where rheumatologists can prescribe disease-modifying therapy, including biological and targeted therapies at their own discretion. We also investigated whether the individual components of the Disease Activity Score 28 (DAS28-CRP) and visual analogue scale (VAS) pain differed by perceived social support and low decision latitude at work.

## Methods

### Study population

Information on psychosocial conditions was retrieved from the Epidemiological Investigation of Rheumatoid Arthritis (EIRA) study. EIRA is a population-based case-control study covering the middle and southern parts of Sweden and includes cases of RA diagnosed by rheumatology specialists within 1 year from diagnosis that meet the American College of Rheumatology (ACR) 1987 and/or 2010 European League Against Rheumatism (EULAR)/ACR criteria for RA [1, 14]. The mean time from first symptom to diagnosis was 10 months. During 1996–2015, 3724 cases and 5935 controls participated in the study. As described in detail elsewhere, the study participants answered a detailed questionnaire regarding their socioeconomic conditions and lifestyle habits [7]. Incomplete responses from the participants were followed up through telephone interviews. The response rate to the study questionnaire was 93% for cases.

### Exposures

An index on the level of social support outside work at the time of RA diagnosis was calculated based on four questions developed by Henderson [15] and Undén and Orth-Gomer [16], with a score range of 4 to 20. Similarly, an index for the level of decision latitude at work was based on six questions (Supplementary Tables S1 and S2) developed by Karasek and Theorell, which focused on influence on the working situation, demands on occupational skills, and possibility of further education in the workplace, with a score range of 6 to 24 [9]. Questions on decision latitude at work were only included during the first decade of the EIRA study (part 1, 1996–2006, 1998 cases); furthermore, only individuals active on the labour market were asked to respond to these questions. Other questions, including questions on social support, were included during a longer EIRA study period (1996–2015, 3724 cases). Other environmental and lifestyle factors were also captured at diagnosis and classified for the present analyses as follows: university degree (yes/no), smoking habits (ever smoking, yes/no), alcohol habits (ever drinking, yes/no), and symptom duration at inclusion (categorized in quartiles). Rheumatoid factor (RF) status was determined using standard procedures at each clinic and of anti-citrullinated protein antibody (ACPA) positivity as the presence of anti-citrullinated peptide (anti-CCP) antibodies as assessed by standard enzyme-linked immunosorbent assay (anti-CCP2 assay, Immunoscan-RA Mark 2 ELISA test; Euro-Diagnostica, Malmö, Sweden).

### Outcome

Information on the outcome, RA disease activity during follow-up, was retrieved from the Swedish Rheumatology Quality register (SRQ), a clinical quality register used to collect data from follow-up visits during routine care. Patient characteristics are registered at diagnosis, and information on disease activity, patient-reported outcome measures (PROMs), and treatment is registered at diagnosis and subsequent follow-up visits. The national coverage of patients with RA in the register is around 86% [17]. Information from SRQ was available until 2020, allowing a follow-up time of 60 months from diagnosis for all patients in the study.

Information on disease activity, defined according to the DAS28-CRP and its components, was captured from the SRQ from calendar years 1996 to 2020 at visits 3 (±2) months, 12 (±3) months, and 60 (±12) months after inclusion. Since each individual might have more than one visit within each timespan, we selected the visit occurring closest to the pre-set time.

Remission at each time point was defined as a DAS28-CRP ≤ 2.4 at the recorded visit in SRQ [18]. The patients’ assessments of pain (VAS pain) were also retrieved from the SRQ and unacceptable pain was defined as a VAS pain >40 [19].

### Statistical analyses

We categorized the main exposures using sex-specific quartiles of the scores among the controls to define the cut-off levels (frequency tables available in Supplementary Tables S3-S6) where the cut-off for the lowest quartiles identified the exposures as compared to the remaining three quartiles. Data from controls were used to avoid possible influence from the RA disease.

Baseline characteristics are presented as medians and interquartile range (IQR).

Logistic regression was used to estimate whether low social support or low decision latitude at work was associated with DAS28-CRP remission at 3, 12, and 60 months, first after adjusting only for age and sex and subsequently with additional adjusting for smoking habits, alcohol habits, symptom duration, and educational level. Additional analyses investigating a possible association between the two main exposures and DAS28-CRP remission were made where the two main exposures were treated as continuous variables instead of dichotomous variables, both in a crude model and in a fully adjusted model.

In a sensitivity analysis, the odds ratio (OR) for remission among patients in the lowest quartiles was compared to the highest quartiles of the exposures. An additional sensitivity analysis was performed, comparing the odds for remission among patients with both low social support and low decision latitude at work as compared with patients without either of these exposures.

Additional analyses for the association between social support and remission were conducted in subgroups based on sex, ACPA status, smoking habits, educational level, alcohol habits, and symptom duration.

The medians of the separate components of the DAS28-CRP as well as of VAS pain were compared between patients with low and not low social support and subsequently for patients with low and not low decision latitude at work. In complementary analyses, the OR for having VAS pain above median for each time point for individuals reporting low social support as compared to not low social support was investigated. The association was tested using logistic regression in a model adjusted for age and sex and further in a fully adjusted multivariable model. Further, the OR for having VAS pain above median for each time point was investigated for individuals reporting low decision latitude at work as compared to not low decision latitude at work, with the same method. Subsequently, in models for the OR of having unacceptable pain (VAS pain >40 mm) at each time point, social support and decision latitude at work, respectively, were investigated, first in a model adjusted for age and sex and further in a fully adjusted model.

The frequencies of the exposures in individuals lost to follow-up and individuals still in the study after 5 years were also compared.

As the rate of missingness for the exposure was low, complete case analyses were performed. Individuals lacking data on social support and individuals without data on decision latitude at work mainly due to incomplete questionnaires were excluded. Missingness for the covariates did not exceed 5%.

Statistical analyses were performed using SAS 9.4 (SAS Institute, Cary, NC, USA). All tests were two-sided and the significance level was set to 0.05. Two-sided *p*-values were calculated using median two-sample tests.

## Results

During 1996–2015, EIRA included 3727 patients, 2820 of whom were also included in the SRQ. Among these, 2800 responded to the questions on social support, of whom 591 were considered to have low social support. The questions on decision latitude at work (only in questionnaire years 1996–2006) were answered by 847 patients (in EIRA part one and active in the labour market), of whom 212 were considered to have low decision latitude at work. The baseline characteristics for individuals with information from both EIRA and SRQ are presented in Table 1.

**Table 1:** Baseline characteristics for patients with rheumatoid arthritis and information from EIRA (1996–2015) and SRQ (*n*=2820)

	All cases, *n*=2820	Subgroup with data on social support, ***n***=2800		Subgroup with data on decision latitude at work, ***n***=847^a^	
		Low social support, *n* = 591	Not low social support, *n* = 2209	Low decision latitude at work, *n* = 212	Not low decision latitude at work, *n* = 635
**Age (years), median (IQR)**	55 (44–62)	56 (46–63)	55 (43–62)	51 (41–57)	52 (42–57)
**Female sex,*n*(%)**	2001 (71.1)	427 (72.3)	1563 (70.8)	151 (71.6)	431 (67.9)
**RF pos,*n*(%)**	1796 (63.9)	374 (63.7)	1406 (63.8)	137 (64.9)	429 (67.6)
**ACPA pos,*n*(%)**	1804 (65.3)	380 (65.4)	1408 (65.2)	138 (66.7)	427 (68.1)
**Symptom duration (months), median (IQR)**	5.8 (3.6–9.4)	5.8 (3.5–9.5)	5.8 (3.6–9.4)	6.0 (4.1–8.9)	5.9 (3.7–8.9)
**Low social support,*n*(%)**	591 (21.0)	-	-	50 (23.7)	103 (16.2)
**Low decision latitude at work,*n*(%)**^a^	211 (25.0)	50 (32.7)	161 (23.2)	-	-
**DAS28-CRP, median (IQR)**	4.9 (4.2–5.7)	5.0 (4.3–5.8)	4.9 (4.1–5.6)	5.1 (4.3–5.9)	5.0 (4.3–5.7)
**HAQ-score, median (IQR)**	1.00 (0.63–1.38)	1.00 (0.75–1.50)	1.00 (0.50–1.38)	1.00 (0.63–1.38)	1.00 (0.50–1.38)
**VAS pain, median (IQR)**	51 (32–70)	55 (35–75)	51 (32–69)	50 (33–68)	49 (29–66)
**VAS global, median (IQR)**	51 (30-69)	55 (35-75)	50 (29–68)	51 (29–69)	49 (27–68)
**CRP, median (IQR)**	12 (6–29)	12 (6–29)	12 (6–28)	14 (8–29)	15 (8–31)
**Swollen joint count (SJC), median (IQR)**	8 (5–12)	8 (5–13)	8 (5–12)	9 (5–14)	9 (5–12)
**Tender joint count (TJC), median (IQR)**	7 (4–12)	8 (4–12)	7 (3–12)	8 (4–13)	8 (4–12)
**Ever smoking,*n*(%)**	1802 (66.4)	408 (71.1)	1380 (65.1)	153 (72.5)	430 (67.7)
**University degree,*n*(%)**	701 (25.9)	100 (17.4)	598 (28.2)	29 (13.7)	225 (35.4)
**Ever drinker,*n*(%)**	2442 (90.3)	491 (86.0)	1937 (91.5)	187 (88.6)	581 (91.5)

Missing data: RF, *n* = 10 (0.4%); ACPA, *n* = 59 (2.1%); symptom duration, *n* = 112 (4.0%); social support, *n* = 20 (0.7%); DAS 28-CRP, *n* = 195 (6.9%); HAQ, *n* = 180 (6.4%); VAS pain, *n* = 141 (5.0%); VAS global, *n* = 130 (4.6%); CRP, *n* = 62 (2.2%); SJC, *n* = 58 (2.1%); TJC, *n* = 57 (2.0%); ever smoking, *n* = 107 (3.8%); educational level, *n* = 108 (3.8%); alcohol habits, *n* = 115 (4.1%)

^a^Information on occupational conditions only available in EIRA 1 (*n* = 1998) which was run 1996–2006

At 3 months’ follow-up, 28% of the patients had achieved DAS28-CRP remission; the corresponding percentages for the 12- and the 60-month follow-up visits were 42% and 50%, respectively.

Neither low social support nor low decision latitude at work was associated with DAS28-CRP remission at any of the follow-up time-points (Table 2).

**Table 2:** Association between social support and decision latitude at work, respectively, and DAS28-CRP remission in patients with rheumatoid arthritis

		DAS28-CRP remission	
	Follow-up time point	OR^a^(95% CI)	OR^b^(95% CI)
**Low vs. not low social support**	3 months	0.86 (0.69–1.07)	0.93 (0.74–1.16)
	12 months	0.89 (0.73–1.10)	0.96 (0.75–1.23)
	60 months	0.89 (0.72–1.10)	0.89 (0.72–1.10)
**Low vs. not low decision latitude**	3 months	0.75 (0.48–1.15)	0.84 (0.54–1.31)
	12 months	0.79 (0.55–1.12)	0.81 (0.56–1.16)
	60 months	1.20 (0.83–1.74)	1.37 (0.94–2.01)

^a^Model adjusted for age and sex

^b^Model further adjusted for smoking habits, educational level, alcohol habits, and symptom duration

There was no significant association between low social support and low decision latitude at work, respectively, and DAS28-CRP remission in sensitivity analyses in which the main exposures were included as continuous instead of binary variables.

Furthermore, changing the reference from the not low social support to the highest quartile of social support did not change the results (Supplementary Table S7). Analysing the association between social vulnerability and DAS28-CRP remission in individuals exposed to both low social support and low decision latitude at work (*n* = 50) compared with individuals without any of these exposures (*n* = 582) did not result in any significant associations (Supplementary Table S8). Analyses of subgroups defined by the adjustment variables showed no association between level of social support and DAS28-CRP remission in any of the subgroups except for the follow-up at 60 months among never-drinkers for which a statistically significant association was observed (Table 3). The never-drinker subgroup at 60 months, however, included the lowest number of respondents of all subgroups (Table 3).

**Table 3:** Associations between low social support and DAS28-CRP remission in patients with rheumatoid arthritis (*n* = 2800); overall and analyses of subgroups

	3 months’ follow-up		12 months’ follow-up		60 months’ follow-up	
	Crude model^a^	Adjusted model^a^	Crude model^a^	Adjusted model^a^	Crude model^a^	Adjusted model^a^
**All patients,*n***	500/1894		479/1838		426/1628	
OR (95% CI)	0.86 (0.69–1.07)	0.93 (0.74–1.16)	0.89 (0.73–1.10)	0.96 (0.77–1.18)	0.89 (0.72–1.10)	0.89 (0.72–1.10)
**Women,*n***	354/1338		341/1294		315/1168	
OR (95% CI)	0.86 (0.66–1.27)	0.93 (0.71–1.22)	0.93 (0.73–1.18)	0.99 (0.77–1.28)	0.88 (0.69–1.13)	0.89 (0.69–1.15)
**Men,*n***	146/556		138/541		111/460	
OR (95% CI)	0.82 (0.55–1.24)	0.90 (0.59–1.37)	0.80 (0.54–1.16)	0.86 (0.58–1.27)	0.89 (0.59–1.36)	0.91 (0.59–1.39)
**ACPA-positive,*n***	322/1210		311/1189		309/1120	
OR (95% CI)	0.89 (0.68–1.17)	0.96 (0.72–1.26)	0.88 (0.68–1.14)	0.92 (0.71–1.19)	0.93 (0.72–1.20)	0.94 (0.73–1.21)
**ACPA-negative,*n***	169/639		162/605		111/482	
OR (95% CI)	0.80 (0.54–1.20)	0.89 (0.59–1.35)	0.93 (0.65–1.32)	1.00 (0.69–1.46)	0.77 (0.51–1.17)	0.79 (0.52–1.21)
**Ever smokers,*n***	350/1182		338/1150		294/1026	
OR (95% CI)	0.82 (0.62–1.07)	0.86 (0.65–1.13)	0.89 (0.69–1.14)	0.93 (0.72–1.20)	0.97 (0.75–1.26)	1.02 (0.78–1.32)
**Never smokers,*n***	136/637		128/615		124/557	
OR (95% CI)	1.09 (0.73–1.63)	1.14 (0.76–1.71)	1.03 (0.70–1.52)	1.05 (0.71–1.55)	0.78 (0.52–1.15)	0.80 (0.54–1.19)
**Education ≤12 years,*n***	399/1288		384/1278		342/1138	
OR (95% CI)	0.89 (0.69–1.15)	0.93 (0.72–1.20)	0.94 (0.74–1.18)	0.97 (0.76–1.23)	0.94 (0.74–1.20)	0.95 (0.74–1.21)
**Education >12 years,*n***	87/530		82/487		76/445	
OR (95% CI)	0.88 (0.54–1.45)	0.91 (0.55–1.51)	0.89 (0.56–1.43)	0.93 (0.58–1.51)	0.92 (0.56–1.51)	0.98 (0.59–1.62)
**Ever drinkers,*n***	417/1665		399/1608		357/1445	
OR (95% CI)	0.94 (0.74–1.19)	0.97 (0.76–1.19)	0.90 (0.72–1.12)	0.92 (0.74–1.15)	1.03 (0.82–1.30)	1.09 (0.86–1.38)
**Never drinkers,*n***	66/152		65/155		58/137	
OR (95% CI)	0.62 (0.30–1.31)	0.61 (0.29–1.31)	1.22 (0.67–2.23)	1.31 (0.71–2.45)	0.34 (0.17–0.66)	0.35 (0.18–0.68)
**Symptom duration above median,*n***	258/976		241/952		207/810	
OR (95% CI)	0.82 (0.60–1.12)	0.88 (0.64–1.21)	0.89 (0.66–1.18)	0.97 (0.72–1.31)	0.96 (0.71–1.30)	1.03 (0.76–1.41)
**Symptom duration below median,*n***	242/918		238/883		219/818	
OR (95% CI)	0.92 (0.67–1.27)	1.01 (0.73–1.40)	0.92 (0.69–1.24)	0.98 (0.72–1.32)	0.82 (0.61–1.11)	0.82 (0.61–1.11)

^a^Logistic models comparing the odds of remission among patients reporting social support in the lowest quartile compared with patients reporting higher social support. Crude model adjusted for age and sex; adjusted model adjusted for age, sex, smoking habits, educational level, alcohol habits, and symptom duration. “n” indicates the number of patients with low social support/not low social support in each subgroup

With respect to objective DAS28-CRP components, none of these showed any statistically significant differences between individuals reporting low social support vs. individuals not reporting low social support. However, worse results of patient-reported outcomes regarding VAS pain scores at 12 and 60 months’ and VAS global scores at the 60 months’ follow-up were observed for individuals reporting low social support as compared to individuals with better social support (Table 4).

**Table 4:** Components of DAS28-CRP and VAS pain in RA-patients reporting low or not-low social support

Follow-up time point	3 months		12 months		60 months	
	Low Social support (***n*** = 500)	Not low social support (***n*** = 1894)	Low Social support (***n*** = 479)	Not low social support (***n*** = 1835)	Low Social support (***n*** = 426)	Not low social support (***n*** = 1628)
**CRP, median (IQR)**	7 (3–10)	7 (3–11)	5 (2–9)	6 (2–9)	3 (1–7)	4 (1–8)
**Tender joint count, median (IQR)**	2 (0–6)	2 (0–5)	1 (0–4)	1 (0–3)	1 (0–3)	0 (0–3)
**Swollen joint count, median (IQR)**	2 (0–5)	2 (0–5)	1 (0–3)	1 (0–2)	0 (0–2)	0 (0–2)
**VAS global, median (IQR)**	30 (13–52)	27 (10–49)	25 (10–54)	21 (7–44)	29 (8–51)*	21 (6–45)*
**VAS pain, median (IQR)**	26 (10–50)	25 (10–48)	25 (9–53)*	21 (6–45)*	27 (9–50)*	22 (6–45)*

*p < 0.05

Similarly, no differences regarding objective measures or VAS pain between patient groups based on decision latitude at work. However, worse status for VAS global scores at 12 months was observed for individuals reporting low decision latitude at work as compared to those in individuals with not low decision latitude at work (Table 5).

**Table 5:** Components of DAS28-CRP and VAS pain in RA-patients reporting low or not low decision latitude at work

	Follow-up time point					
	3 months		12 months		60 months	
	Low decision latitude at work (***n*** = 187)	Not low decision latitude at work (***n*** = 563)	Low decision latitude at work (***n*** = 189)	Not low decision latitude at work (***n*** = 568)	Low decision latitude at work (***n*** = 153)	Not low decision latitude at work (***n*** = 491)
**CRP, median (IQR)**	8 (6–14)	9 (7–15)	8 (4–10)	8 (5–10)	4 (2–8)	5 (2–8)
**Tender joint count, median (IQR)**	3 (1–7)	2 (0–6)	2 (0–5)	1 (0–4)	1 (0–3)	1 (0–3)
**Swollen joint count, median (IQR)**	3 (1–7)	3 (0–7)	1 (0–4)	1 (0–3)	1 (0–3)	0 (0–2)
**VAS global, median (IQR)**	35 (15–53)	29 (13–50)	27 (11–56)*	21 (7–44)*	21 (8–46)	23 (7–44)
**VAS pain, median (IQR)**	30 (15–53)	26 (13–48)	26 (12–51)	20 (6–46)	25 (9–45)	22 (7–45)

*p < 0.05

In the logistic regression model investigating the association between low social support (and low decision latitude at work, respectively) and VAS pain above median at each follow-up time point, there was an association between low social support and pain above median at the 12- and 60-month follow-up visits. Furthermore, low decision latitude at work was associated with pain above median at the 12-month follow-up visit. The observed association at 60 months remained statistically significant in the fully adjusted model (Table 6). The corresponding analyses for VAS global showed an association between low social support and VAS global above median at 60 months and also between low decision latitude and VAS global above median at 12 months. Both observed associations remained after further adjustments (Table 6).

**Table 6:** Associations between social support and decision latitude at work, respectively, and patient-reported outcome measures

		VAS pain above median^**1**^		VAS global above median^**2**^	
	Follow-up time point	OR*(95% CI)	OR**(95% CI)	OR*(95% CI)	OR**(95% CI)
**Low social support**	3 months	1.12 (0.92–1.37)	0.99 (0.81–1.22)	**1.26 (1.03**–**1.53)**	1.17 (0.95–1.44)
	12 months	**1.30 (1.06**–**1.59)**	1.22 (0.99–1.50)	1.20 (0.98–1.47)	1.12 (0.91–1.38)
	60 months	**1.34 (1.08**–**1.66)**	**1.26 (1.01**–**1.57)**	**1.40 (1.13**–**1.74)**	**1.35 (1.08**–**1.68)**
**Low decision latitude at work**	3 months	1.29 (0.92–1.80)	1.12 (0.79–1.59)	1.32 (0.94–1.85)	1.21 (0.861.71)
	12 months	**1.46 (1.05**–**2.03)**	1.34 (0.95–1.88)	**1.53 (1.10**–**2.14)**	**1.45 (1.03**–**2.04)**
	60 months	1.10 (0.76–1.60)	1.05 (0.72–1.54)	0.94 (0.65–1.35)	0.87 (0.60–1.27)

^1^Median for VAS pain at each time-point: 3 months 25 mm, 12 months 22 mm, 60 months 24 mm

^2^Median for VAS global at each time-point: 3 months 28 mm, 12 months 22 mm, 60 months 23 mm

*Adjusted for age and sex. **Adjusted for age, sex, smoking, alcohol use, educational level, and symptom duration**.** Statistically significant results in bold

No significant association between low social support and unacceptable pain (VAS pain>40) at any of the time points was observed (Supplementary Table S9).

Individuals lost to follow-up at 60 months (*n* = 755, 26.8 %) were more often men, older, seronegative, and ACPA-negative and had lower baseline values of VAS pain score, VAS global score, and CRP. However, there were no statistically significant differences in the frequency of those lost to follow-up between the two main exposures (Supplementary Table S10).

## Discussion

In this large, observational study of patients with newly diagnosed RA in a setting with general access to healthcare, no association was observed between psychosocial vulnerability (defined as low social support and low decision latitude at work) and remission during the first 5 years from RA diagnosis.

The findings of the present study support those of a previous study on socioeconomic factors based on the EIRA-cohort, that defined socioeconomic status using educational level [13]. In that study, educational level did not influence the disease activity after 1 year; however, individuals with a lower educational level reported more pain at the 12-month follow-up. In another previous study, we observed an association between educational level and social support [20], which may explain the tendency towards higher levels of pain in patients reporting low social support observed in our present study.

In a study from Great Britain that defined socioeconomic status based on residential area, low socioeconomic status was associated with an increased risk of a refractory RA disease [11]. Socioeconomic status was defined based on social deprivation scores for England, Scotland, and Wales, while refractory disease was defined as exposure to at least three different classes of biologic treatments. In a recent study from the Netherlands, low socioeconomic status, defined as lower educational level, was independently associated with the risk of developing a difficult-to-treat RA, as defined by the European Alliance of Associations for Rheumatology (formerly the European League Against Rheumatism, EULAR) [21, 22]. However, it is difficult to compare these results to those from the present study since the outcome measures were not the same and since the studies were conducted in different settings.

It cannot be ruled out that modern treatment strategies for RA could weaken or diminish the possible influence of RA disease risk factors, such as socioeconomic strain, on disease outcome. Such an effect was previously observed in the BeSt study for ACPA, rheumatoid factor and genetic risk factors in relation to radiographic progression in early RA [23]. Our study was performed in a setting with general access to healthcare irrespective of socioeconomic level. Furthermore, the large number of patients, the high response rate, and the long follow-up time all add to the clinical relevance and generalizability. The real-life setting with information on outcome from the clinical quality register with high coverage in Sweden is an advantage, minimizing (but not excluding) the risk for selection bias where individuals with low social support and/or low decision latitude in EIRA are less represented in the SRQ. Individuals with severe psychosocial strain may have been less well represented in the EIRA and the most psychosocially vulnerable patients might not have been included in EIRA in the first place.

The concept of psychosocial vulnerability is complex and not easily captured in a questionnaire, ours included. However, these exposures were associated with other characteristics of low socioeconomic status such as smoking and educational level [20], defining groups with clusters of risk factors that might influence the outcome. Moreover, decision latitude at work could only be assessed from the questions used during the first decade of the EIRA study and only applied to patients active in the labour market; thus, data were only available for a smaller subgroup of participants. Anti-rheumatic treatment during the five-year follow-up was not taken into consideration in this study. Hence, whether remission was achieved through different means in individuals with vs. without psychosocial vulnerability, or if similar therapeutic strategies were used irrespectively of socioeconomic factors, remains unknown. A previous study from the EIRA study, with a 12-month follow-up, indicated no difference in treatment between individuals with low vs. higher socioeconomic status [13]. Also, whether individuals with psychosocial vulnerability have a higher risk of difficult to treat RA, with multiple changes of therapy, is a question for further studies.

In the study, we observed a tendency for individuals with low social support and low decision latitude at work, respectively, to report worse health status on the subjective measures, VAS pain and VAS global health. This tendency was not uniform or fully independent of confounders, but was observed in several of the analyses.

## Conclusion

The results of this study indicate that psychosocial vulnerability despite being a risk factor for development of RA [6, 7] does not influence the likelihood of achieving remission for patients with RA in the Swedish setting, with equal access to healthcare.

## Supplementary Information

**Additional file 1: Table S1**. Description of the questions on social support. **Table S2**. Description of the questions on decision latitude at work. **Table S3**. Frequency-table for social support among female controls. Cut off level for lowest quartile indicated with a horizontal line. **Table S4**. Frequency table for social support among male cases and controls. Cut off level for lowest quartile indicated with a horizontal line. **Table S5**. Frequency table for decision latitude at work among female cases and controls. Cut off level for lowest quartile indicated with a horizontal line. **Table S6**. Frequency table for decision latitude at work among male cases and controls. Cut off level for lowest quartile indicated with a horizontal line. **Table S7**: Association of social support and decision latitude at work with DAS28-CRP remission at different time points. **Table S8**: Association of social support and decision latitude at work with DAS28-CRP remission at different time points comparing individuals with both low social support and low decision latitude (*n*=50) with individuals with not low social support and not low decision latitude (*n*=582). **Table S9**. Odds ratios for VAS pain >40 mm at different time points in patients with low vs not low social support and decision latitude at work, respectively. **Table S10**. Comparison of baseline characteristics of individuals lost to follow-up and individuals still in study on follow-up at 60 months’ follow-up. Table including all adjusting variables.

## Data Availability

The data that support the findings of this study are available from Karolinska Institutet but restrictions apply to the availability of these data, which were used under licence for the current study, and so are not publicly available. Data are however available from the authors upon reasonable request and with permission of Karolinska Institutet.

## Abbreviations

ACPA: Anti-citrullinated protein antibody
ACR: American College of Rheumatology
CRP: C-reactive protein
DAS28: Disease Activity Score with 28 joint count
EIRA: Epidemiological Investigation in Rheumatoid Arthritis
EULAR: European League Against Rheumatism
IQR: Interquartile range
OR: Odds ratio
PROM: Patient-reported outcome measure
RA: Rheumatoid arthritis
RF: Rheumatoid factor
SRQ: Swedish Rheumatology Quality Register
VAS: Visual analogue scale
