# Supplementary material for: Neither low social support nor low decision latitude at work is associated with disease remission among patients with rheumatoid arthritis: results from the Swedish EIRA study
Source: Arthritis Res Ther. 2022 Aug 23;24:203. doi: 10.1186/s13075-022-02892-w (PMC9396875; doi:10.1186/s13075-022-02892-w)
Supplement: Supplementary file 1 — Additional file 1: Table S1. Description of the questions on social support. Table S2. Description of the questions on decision latitude at work. Table S3. Frequency-table for social support among female controls. Cut off level for lowest quartile indicated with a horizontal line. Table S4. Frequency table for social support among male cases and controls. Cut off level for lowest quartile indicated with a horizontal line. Table S5. Frequency table for decision latitude at work among female cases and controls. Cut off level for lowest quartile indicated with a horizontal line. Table S6. Frequency table for decision latitude at work among male cases and controls. Cut off level for lowest quartile indicated with a horizontal line. Table S7: Association of social support and decision latitude at work with DAS28-CRP remission at different time points. Table S8: Association of social support and decision latitude at work with DAS28-CRP remission at different time points comparing individuals with both low social support and low decision latitude (n=50) with individuals with not low social support and not low decision latitude (n=582). Table S9. Odds ratios for VAS pain >40 mm at different time points in patients with low vs not low social support and decision latitude at work, respectively. Table S10. Comparison of baseline characteristics of individuals lost to follow-up and individuals still in study on follow-up at 60 months’ follow-up. Table including all adjusting variables. [file 13075_2022_2892_MOESM1_ESM.docx]

**Supplementary**

**Contents**

Description of the questions on social support work (S1) …………………….…………………Page 2

Description of the questions on decision latitude at work (S2)………………………………Page 3

Frequency-table for social support among female cases and controls (S3)……………..Page 4

Frequency-table for social support among male cases and controls (S4)………..……...Page 5

Frequency-table for decision latitude at work among

female cases and controls (S5)……………………………………………………………….….…………..Page 6

Frequency-table for decision latitude at work among

male cases and controls (S6)……………………………………………………………….………………...Page 7

Sensitivity analyse with ORs calculated with

highest quartile as reference (S7)……………………………………………………………………………..Page 8

Sensitivity analyse with ORs calculated on individuals with

both low social support and low social support vs individuals with not low social

support and not low decision latitude (S8)……………………………………………………………… Page 8

Sensitivity analyse with ORs for reported pain above 40 mm at

each time-point (S9)…………………………………………………………………………………………………Page 9

Characteristics of individuals lost to follow up (S10)………………………………………………....Page 10

**Table S1. Description of the questions on social support**

**Questions on social support:**

1. How many individuals with the same interests as you do you know and spend time with?

| 1. Outside work 2. At work |
| --- |

1. How many of your friends and family can you speak to freely, openly and honestly without having to first think about what you are saying?

| 1. Outside work 2. At work |  |
| --- | --- |

1. Besides the people at home, there are others to whom I can turn if I have problems – people I can easily meet, whom I trust, and who can really help me when I am in trouble.

| 1. Outside work 2. At work |
| --- |

1. I feel a strong sense of belonging with

| 1. My extended family (in addition to spouse/child) 2. My colleagues at work   Answer options and grading of questions 1-2:  Nobody (1p)  1-2 person(s)(2p)  3-5 people (3p)  6-10 people (4p)  11-15 people (5p)  more than 15 people (6p)  Answer options and grading of questions 3-4:  How well do the following statements apply to you?  Strongly agree (4p)  Agree somewhat (3p)  Disagree somewhat (2p)  Strongly disagree (1p) |  |
| --- | --- |
| We decided to analyse the questions concerning social support outside work |  |

Minimum points for social support with this setting was 4 and the maximum points was 20, where a higher numerical value indicates higher sense of social support.

Women scoring ≤ 11 and men scoring ≤10 are considered exposed. The quartiles were calculated on controls.

**Table S2. Description of the questions on decision latitude at work**

**Questions on decision latitude (control) at work.**

1. Are there opportunities to learn new things at work?
2. Does your work demand working-skills?
3. Does your work demand inventiveness?
4. Do you have the freedom to decide how your work will be/was done?
5. Do you have the freedom to decide what should be done in your work?
6. Do you have to do the same thing over and over again at your work?

Answer options and grading of questions 1-5:

- Yes, often (4 points)
- Yes, sometimes (3 points)
- No, seldom (2 points)
- No, almost never (1 point)

Answer options and grading of question 6:

- Yes, often (1 point)
- Yes, sometimes (2 points)
- No, seldom (3 points)
- No, almost never (4 points)

Minimum points for decision latitude at work was 6 and the maximum points was 24, where a higher numerical value indicates higher decision latitude. Women scoring ≤ 16 and men scoring ≤17 are considered exposed. The quartiles were calculated on controls.

**Table S3. Frequency-table for social support among female controls. Cut off level for lowest quartile indicated with a horizontal line.**

|  | **Cases** |  | **Controls** |  |
| --- | --- | --- | --- | --- |
| **Social** | **Frequency** | **Cumulative percent** | **Frequency** | **Cumulative percent** |
| 4 | 4 | 0.15 | 11 | 0.26 |
| 5 | 18 | 0.83 | 21 | 0.76 |
| 6 | 23 | 1.69 | 21 | 1.26 |
| 7 | 35 | 3.01 | 36 | 2.11 |
| 8 | 39 | 4.48 | 83 | 4.08 |
| 9 | 85 | 7.68 | 129 | 7.14 |
| 10 | 151 | 13.36 | 217 | 12.28 |
| 11 | 222 | 21.72 | 349 | 20.56 |
| 12 | 282 | 32.33 | 444 | 31.09 |
| 13 | 370 | 46.26 | 549 | 44.11 |
| 14 | 381 | 60.59 | 585 | 57.98 |
| 15 | 306 | 72.11 | 517 | 70.24 |
| 16 | 247 | 81.41 | 434 | 80.53 |
| 17 | 163 | 87.54 | 306 | 87.79 |
| 18 | 164 | 93.71 | 243 | 93.55 |
| 19 | 83 | 96.84 | 143 | 96.94 |
| 20 | 84 | 100.00 | 129 | 100.00 |
|  |  |  |  |  |

Frequency missing: cases n=19, controls n=33

**Table S4. Frequency table for social support among male cases and controls. Cut off level for lowest quartile indicated with a horizontal line.**

|  | **Cases** | | **Controls** | |
| --- | --- | --- | --- | --- |
| **Social** | **Frequency** | **Cumulative percent** | **Frequency** | **Cumulative percent** |
| 4 | 7 | 0.67 | 7 | 0.42 |
| 5 | 7 | 1.34 | 7 | 0.84 |
| 6 | 9 | 2.21 | 22 | 2.16 |
| 7 | 19 | 4.03 | 32 | 4.07 |
| 8 | 39 | 7.77 | 56 | 7.43 |
| 9 | 63 | 13.81 | 84 | 12.46 |
| 10 | 72 | 20.71 | 139 | 20.79 |
| 11 | 105 | 30.78 | 167 | 30.80 |
| 12 | 108 | 41.13 | 178 | 41.46 |
| 13 | 136 | 54.17 | 207 | 53.86 |
| 14 | 118 | 65.48 | 205 | 66.15 |
| 15 | 119 | 76.89 | 187 | 77.35 |
| 16 | 72 | 83.80 | 129 | 85.08 |
| 17 | 59 | 89.45 | 99 | 91.01 |
| 18 | 50 | 94.25 | 64 | 94.85 |
| 19 | 24 | 96.55 | 43 | 97.42 |
| 20 | 36 | 100.00 | 43 | 100.00 |
|  |  |  |  |  |
|  |  |  |  |  |
|  |  |  |  |  |
|  |  |  |  |  |
|  |  |  |  |  |

Frequency missing: cases n=5, controls n=16

**Table S5. Frequency table for decision latitude at work among female cases and controls. Cut off level for lowest quartile indicated with a horizontal line.**

|  | **Cases** | | **Controls** | |
| --- | --- | --- | --- | --- |
| **Decision latitude at work** | **Frequency** | **Cumulative percent** | **Frequency** | **Cumulative percent** |
| 6 | 2 | 0.22 |  |  |
| 7 | 1 | 0.34 | 3 | 0.28 |
| 8 | 2 | 0.56 | 2 | 0.46 |
| 9 |  |  | 3 | 0.74 |
| 10 | 7 | 1.34 | 7 | 1.38 |
| 11 | 9 | 2.35 | 8 | 2.12 |
| 12 | 15 | 4.03 | 14 | 3.40 |
| 13 | 27 | 7.05 | 24 | 5.61 |
| 14 | 47 | 12.32 | 39 | 9.20 |
| 15 | 46 | 17.47 | 55 | 14.26 |
| 16 | 74 | 25.76 | 83 | 21.90 |
| 17 | 95 | 36.39 | 107 | 31.74 |
| 18 | 94 | 46.92 | 107 | 41.58 |
| 19 | 119 | 60.25 | 144 | 54.83 |
| 20 | 127 | 74.47 | 148 | 68.45 |
| 21 | 110 | 86.79 | 143 | 81.60 |
| 22 | 81 | 95.86 | 111 | 91.81 |
| 23 | 30 | 99.22 | 68 | 98.07 |
| 24 | 7 | 100.00 | 21 | 100.00 |
|  |  |  |  |  |
|  |  |  |  |  |
|  |  |  |  |  |
|  |  |  |  |  |

Frequency missing: cases n=528, controls n=506

**Table S6. Frequency table for decision latitude at work among male cases and controls. Cut off level for lowest quartile indicated with a horizontal line.**

|  | **Cases** | | **Controls** | |
| --- | --- | --- | --- | --- |
| **Decision latitude at work** | **Frequency** | **Cumulative percent** | **Frequency** | **Cumulative percent** |
| 6 |  |  | 1 | 0.22 |
| 8 | 3 | 0.77 | 1 | 0.43 |
| 9 |  |  | 3 | 1.08 |
| 10 | 4 | 1.79 | 3 | 1.73 |
| 11 | 1 | 2.05 | 3 | 2.38 |
| 12 | 1 | 2.30 | 5 | 3.46 |
| 13 | 5 | 3.58 | 4 | 4.32 |
| 14 | 5 | 4.86 | 10 | 6.48 |
| 15 | 14 | 8.44 | 14 | 9.50 |
| 16 | 22 | 14.07 | 24 | 14.69 |
| 17 | 40 | 24.30 | 25 | 20.09 |
| 18 | 32 | 32.48 | 39 | 28.51 |
| 19 | 66 | 49.36 | 50 | 39.31 |
| 20 | 62 | 65.22 | 95 | 59.83 |
| 21 | 65 | 81.84 | 77 | 76.46 |
| 22 | 42 | 92.58 | 66 | 90.71 |
| 23 | 21 | 97.95 | 41 | 99.57 |
| 24 | 8 | 100.00 | 2 | 100.0 |
|  |  |  |  |  |
|  |  |  |  |  |
|  |  |  |  |  |
|  |  |  |  |  |

Frequency missing: cases n=186, controls n=196

**Table S7: Association of social support and decision latitude at work with DAS28-CRP remission at different time points.**

| **Low social support (n=591), lowest quartile vs highest quartile (n=802)** | | |
| --- | --- | --- |
|  | OR* (95% CI) | OR** (95% CI) |
| **3 months** | 0.86 (0.67-1.11) | 0.94 (0.72-1.22) |
| **12 months** | 0.87 (0.69-1.11) | 0.96 (0.75-1.23) |
| **60 months** | 0.82 (0.64-1.05) | 0.83 (0.64-1.06) |
| **Low decision latitude, lowest quartile (n=212) vs highest quartile (n=240)** | | |
|  | OR* (95% CI) | OR** (95% CI) |
| **3 months** | 0.73 (0.44-1.21) | 0.88 (0.52-1.50) |
| **12 months** | 0.74 (0.49-1.12) | 0.75 (0.48-1.16) |
| **60 months** | 1.30 (0.84-2.01) | 1.66 (1.04-2.66) |

*Adjusted for age and sex **Further adjusted for smoking habits, educational level, alcohol habits and symptom duration

**Table S8: Association of social support and decision latitude at work with DAS28-CRP remission at different time points comparing individuals with both low social support and low decision latitude (n=50) with individuals with not low social support and not low decision latitude (n=582)**

|  | **Low social support and low decision latitude** | |
| --- | --- | --- |
|  | OR* (95% CI) | OR** (95% CI) |
| **3 months** | 0.77 (0.35-1.72) | 0.93 (0.41-2.12) |
| **12 months** | 0.60 (0.30-1.22) | 0.67 (0.32-1.39) |
| **60 months** | 0.66 (0.32-1.37) | 0.74 (0.35-1.57) |

*Adjusted for age and sex **Further adjusted for smoking habits, educational level, alcohol habits and symptom duration

**Table S9. Odds ratios for VAS pain >40 mm at different time points in patients with low vs not low social support and decision latitude at work, respectively**

|  |  | **VAS pain above 40 mm** | |
| --- | --- | --- | --- |
|  | Follow-up time point | OR* (95%CI) | OR (95% OR)** |
| Low social support | 3 months | 1.22 (0.99-1.51) | 1.10 (0.63-1.38) |
|  | 12 months | 1.32 (0.93-1.85) | 1.20 (0.96-1.50) |
|  | 60 months | 1.24 (0.99-1.56) | 1.18 (0.93-1.49) |
| Low decision latitude at work | 3 months | 1.32 (0.94-1.87) | 1.22 (0.85-1.74) |
|  | 12 months | 1.41 (0.99-2.00) | 1.25 (0.87-1.79) |
|  | 60 months | 1.09 (0.73-1.62) | 1.02 (0.67-1.54) |

*Adjusted for age and sex

** Adjusted for age, sex, smoking, alcohol use, educational level and symptom duration

**Table S10. Comparison of baseline characteristics of individuals lost to follow-up and individuals still in study on follow-up at 60 months’ follow-up. Table including all adjusting variables**

|  | **Lost to follow up at 60 months n=755 (26.8%)** | **Still in study at 60 months**  **n=2065 (73.2%)** | **p-value**** |
| --- | --- | --- | --- |
| **Age (years), median (IQR)** | **57 (45-64)** | **54 (44-62)** | **<0.0001** |
| **Female sex, n (%)** | **495 (65.6)** | **1456 (72.4)** | **<0.0001** |
| **RF pos, n (%)** | **375 (49.7)** | **1359 (67.8)** | **<0.0001** |
| **ACPA pos, n (%)** | **346 (45.8)** | **1391 (70.3)** | **<0.0001** |
| **Symptom duration (days), median (IQR)** | 165 (105-278) | 177 (108-286) | 0.30 |
| **Low social support** | 160 (21.2) | 413 (20.7) | 0.35 |
| **Low decision latitude at work*** | 57 (29.2) | 152 (23.8) | 0.13 |
| **DAS28-CRP, median (IQR)** | 4.8 (4.1-5.6) | 4.9 (4.1-5.7) | 0.25 |
| **HAQ-score, median (IQR)** | 1.00 (0.5-1.38) | 1.00 (0.63-1.38) | 0.12 |
| **VAS pain, median (IQR)** | **49 (30-69)** | **53 (33-70)** | **0.003** |
| **VAS global, median(IQR)** | **48 (28-68)** | **52 (30-70)** | **0.02** |
| **CRP, median (IQR)** | **10 (6-26)** | **12 (6-29)** | **0.02** |
| **Swollen joint count (SJC), median (IQR)** | 8 (5-12) | 8 (5-13) | 0.54 |
| **Tender joint count (TJC), median (IQR)** | 7 (3-12) | 7 (4-12) | 0.53 |
| **Ever smoking, n (%)** | 456 (50.4) | 1297 (62.8) | 0.43 |
| **University degree, n (%)** | 170 (22.5) | 502 (25.6) | 0.88 |
| **Ever drinker, n (%)** | 606 (80.3) | 1758 (90.0) | 0.75 |

*Information on working conditions only from EIRA 1

** P-value calculated with median two-sample test, two-sided. Statistically significant differences in bold (p<0.05)
